# A complete season with attendance restrictions confirms the relevant contribution of spectators to home advantage and referee bias in association football

**DOI:** 10.7717/peerj.13681

**Published:** 2022-07-04

**Authors:** Fabrizio Sors, Michele Grassi, Tiziano Agostini, Mauro Murgia

**Affiliations:** Department of Life Sciences, University of Trieste, Trieste, Italy

**Keywords:** Crowd, Football, Officials, Social pressure, Support

## Abstract

**Background:**

Due to the unfortunate pandemic situation, the phenomena of home advantage and referee bias in sports have recently received a particular research attention, especially in association football. In this regard, several studies were conducted on the last portion of the 2019–20 season: the majority of them suggests a reduction—but not the elimination—of the two phenomena, with some exceptions in which no reduction was found or, at the other extreme, the phenomena were not observed at all.

**Methods:**

The continuation of the pandemic made it possible to replicate the previous studies considering the complete 2020–21 season, thus with the important added value of having a fully balanced home/away schedule—and a higher number of matches—in the various leagues. In particular, the sample of the present study consisted of 3,898 matches from the first and second divisions of the UEFA top five ranked countries, that is, England, Spain, Italy, Germany, and France. For the home advantage, the following variables were examined: distribution of matches outcomes and home advantage for points (also for previous seasons from the 2014–15 one); ball possession; total shots; shots on goal; and corner kicks. Instead, for he referee bias, the following variables were examined: fouls; yellow cards; red cards; penalty kicks; and extra time. Chi-square tests were used to compare the distribution of matches outcomes, and t-tests to compare home *vs*. away data for the other variables in the 2020–21 season; Bayesian and equivalence analyses were also conducted.

**Results:**

The main results are as follows: (a) the distribution of matches outcomes in the 2020–21 season was significantly different from that of the last five complete seasons with spectators (Chi-square = 37.42, df = 2, *p* < 0.001), with fewer home victories and more away victories; the resulting values of the home advantage for points were 54.95% for the 2020–21 season, and 59.36% for the previous seasons; (b) for the other home advantage variables, a statistically significant overall advantage for the home team emerged; nevertheless, the strength of the differences between home and away teams was generally small (0.09 < Cohen’s *d* < 0.17), and the corresponding means can be considered statistically equivalent for all variables but the total shots; (c) no statistically significant differences emerged between home and away teams for any of the referee bias variables.

**Discussion:**

These findings demonstrate that the absence of spectators significantly reduced the home advantage compared to previous seasons with spectators. A slight home advantage persisted in the 2020–21 season, probably due to other factors, namely, learning and travel, according to the model by Courneya & Carron (1992). Conversely, the referee bias was not observed, suggesting that it mainly derives from the pressure normally exerted by spectators.

## Introduction

In sport scientific literature, two phenomena have recently received a particular research attention: the *home advantage* and the *subconscious referee bias*. Such phenomena have a long research tradition, starting in the ‘70s with the study of Schwartz & Barsky (1977), and steadily continuing in the ‘80s (*e.g*., Pollard, 1986), ‘90s (*e.g*., Courneya & Carron, 1992), and in the new millennium (*e.g*., Carron, Loughhead & Bray, 2005; Dohmen & Sauermann, 2016; Jamieson, 2010; for a historical overview, see Gómez-Ruano & Pollard, 2022). These and other related studies have consistently documented that professional athletes/teams obtain significantly better results at home than away, also thanks to the more favorable treatment they receive from the referees/officials in the former case. In particular, the sport in which the two phenomena have been studied the most is association football, where the proportion of points won at home is normally higher than the proportion of points won away (Leite & Pollard, 2018; Pollard, 1986, 2006; Pollard & Gomez, 2009, 2014), and referees award fewer sanctions against the home team than against the away team (*e.g*., Goumas, 2014; Nevill, Balmer & Williams, 2002; Sutter & Kocher, 2004; Unkelbach & Memmert, 2010).

In addition to documenting the two phenomena, some researchers also proposed conceptual frameworks to try to explain their causes and the relations among them (see Courneya & Carron, 1992; Pollard, 2006; Pollard & Pollard, 2005). In this regard, Courneya and Carron suggested that the game location factors—crowd, learning, travel, and rules—affect the psychological and behavioral states of athletes, coaches and referees, which in turn influence the performance outcomes. Other studies tried to disentangle the relative “weight” of such factors, and especially that of crowd support, reasonably considered among the most influential ones. Normally, this was done by adopting one of three approaches: (a) analyzing the crowd size, proximity, and/or density (Buraimo, Forrest & Simmons, 2010; Buraimo, Simmons & Maciaszczyk, 2012; Downward & Jones, 2007; Goumas, 2014; Page & Page, 2010); (b) studying a few “unusual matches” like those behind closed doors and/or the same-stadium derbies (Pettersson-Lidbom & Priks, 2010; Ponzo & Scoppa, 2018; van de Ven, 2011); (c) specifically for the referee bias, manipulating the crowd noise in laboratory settings (Balmer et al., 2007; Nevill, Balmer & Williams, 2002; Unkelbach & Memmert, 2010). Such studies—even those characterized by similar approaches—often yielded conflicting results.

The abovementioned particular attention recently received by the home advantage and the referee bias is due to the unfortunate pandemic situation, which is giving the unprecedented opportunity to study large samples of matches played with no or severely limited attendance, allowing for large-scale natural experiments on the effects of the absence of spectators support. An overview of the first studies conducted during the pandemic was provided by Lago-Peñas & Gómez-Ruano (2022), who observed a reduction of both phenomena. This observation was further confirmed in a systematic literature review by Leitner et al. (2022), who identified 26 studies that analyzed the last portion of the 2019–20 season, when all matches were played behind closed doors. Out of these 26 studies, only six did not observe a significant and consistent overall decrease of the home advantage and referee bias in the considered leagues (Almeida & Werlayne, 2021; Benz & Lopez, 2021; Krawczyk & Strawinski, 2020; Matos et al., 2021; Ramchandani & Millar, 2021; Wunderlich et al., 2021), while the other 20 ones did observe a significant decrease of these phenomena (Bryson et al., 2021; Correia-Oliveira & Andrade-Souza, 2021; Cross & Uhrig, 2020; Cueva, 2020; Dilger & Vischer, 2020; Endrich & Gesche, 2020; Ferraresi & Gucciardi, 2020, 2021; Fischer & Haucap, 2020; Hill & Van Yperen, 2021; Jimenez Sanchez & Lavin, 2021; Konaka, 2021; Leitner & Richlan, 2021a; Link & Anzer, 2021; McCarrick et al., 2021; Rovetta & Abate, 2021; Santana, Bettega & Dellagrana, 2021; Scoppa, 2021; Sors et al., 2021; Tilp & Thaller, 2020). Considering exclusively the peer-reviewed, multi-leagues studies, they found that nine out of 13 studies observed such a decrease (Bryson et al., 2021; Correia-Oliveira & Andrade-Souza, 2021; Ferraresi & Gucciardi, 2021; Hill & Van Yperen, 2021; Jimenez Sanchez & Lavin, 2021; Leitner & Richlan, 2021a; McCarrick et al., 2021; Scoppa, 2021; Sors et al., 2021). Based on these findings, Leitner et al. (2022) conclude that spectators support significantly contributes to determine the outcome of matches in professional football.

As mentioned, these studies could only rely on a portion of the 2019–20 season; this means that the home/away schedule of the leagues was not balanced. Aware of this, the majority of the researchers considered several leagues, and included in the analyses some variables to control for the relative strength of the teams playing against each other (*e.g*., points per match ratio), thus ensuring the results an acceptable level of reliability and validity. Due to the continuation of the pandemic, the whole 2020–21 season took place under severe attendance restrictions, with the vast majority of matches played behind closed doors, and the remaining ones with limited attendance. This situation made it possible to replicate the previous studies, but with the important added value of having a fully balanced home/away schedule—and a higher number of matches—in the various leagues. In light of these considerations, the aim of the present study is to investigate whether—and in case how—the home advantage and the referee bias occurred in the domestic leagues during the complete 2020–21 season. Based on recent studies on matches behind closed doors, our hypotheses are: (a) a significant reduction of the home advantage with respect to previous seasons with spectators; (b) a slight occurrence of the home advantage (due to the permanence of learning and travel factors); and (c) no occurrence of the referee bias.

## Materials and Methods

### Sample

The database for the present study was represented by all the 2020–21 regular season’s matches of the first and second divisions of the UEFA top five ranked countries–England, Spain, Italy, Germany, and France; the only matches that were excluded are the same-stadium derbies (six matches, all from the Italian first division) and those matches whose win was awarded by forfeit (two matches, one from the Italian second division and one from the French second division). The final sample consisted of 3,898 matches: 3,535 (90.7% of the sample) were played behind closed doors, that is, in total absence of spectators, while in the remaining 363 (9.3% of the sample) there was an average attendance of 2,937 ± 2,359 spectators^1^ 1In the same leagues, the average attendance in the five seasons before the pandemic (from 2014–15 to 2018–19) was of 20,851 spectators (source: transfermarkt.com).. Moreover, matches outcomes were retrieved also for the last five complete seasons without attendance restrictions (*i.e*., from 2014–15 to 2018–19), as well as for the 2019–20 season (partially played without spectators).

### Variables

The variables taken into consideration are reported in Table 1. Data collection was similar to Sors et al. (2021). In particular, as normally done in previous studies, the data were retrieved from the official websites of the leagues; data that were not available on these websites were retrieved from other trusted ones (*e.g*., bbc.com/sport and soccerway.com). To check for the reliability of the sources, the data of 200 random matches (*i.e*., 20 for each league) were retrieved from two different sources and compared between them; data were highly consistent (average *r* = 0.98).

**Table 1:** Variables considered and their grouping.

Group of variables	Variables
Home advantage variables	Match outcome^a^, Home advantage for points^a^^,^^b^, Goals scored, Ball possession (%), Total shots, Shots on goal, Corner kicks
Referee bias variables	Fouls, Yellow cards^c^, Red cards^d^, Penalty kicks (against), Extra time^e^

**Notes:**

a Also for previous seasons from the 2014–15 one.

b Percentage of points earned by home teams out of the total number of points earned by both home and away teams (*i.e*., Pollard’s traditional method; Matos, Amaro & Pollard, 2020).

c Both first and second yellow cards, issued against players on the pitch within the end of the match.

d Both double-yellow and straight red cards, issued against players on the pitch within the end of the match.

e Minutes of extra time played in the second half of matches whose result at 90’ had a difference of one goal.

### Statistical analysis

The distribution of matches outcomes of the 2020–21 season was compared with the expected frequencies calculated based on the last five complete seasons without attendance restrictions (from 2014–15 to 2018–19) of the same leagues. In particular, we used the Chi-square test to evaluate statistically significant differences among the observed/expected counts of the three mutually exclusive outcomes—home victory, tie, away victory. Moreover, a set of Chi-square tests was carried out season by season to compare the distribution of the 2020–21 matches outcomes with those observed for each single season (from 2014–15 to 2018–19); this was done also for the 2019–20 season, separately for matches with and without spectators. Based on the same data, we calculated the home advantage for points using the Pollard’s traditional method (Matos, Amaro & Pollard, 2020) for each season taken into consideration (and separating matches with and without spectators in the 2019–20 season).

As for the performance-related home advantage variables (goals scored, ball possession, total shots, shots on goal, and corner kicks) and the referee bias variables (fouls, yellow cards, red cards, penalty kicks, and extra time), we performed a paired sample t-test on Home and Away team’s data within each match for each variable. Preliminarily, distribution properties of each variable were inspected by computing asymmetry and kurtosis indices, along with the result of Kolmogorov–Smirnov tests for normality assumption (*p* > 0.05). Based on the expected direction of the effects, the t-tests were one-sided.

To quantify the extent to which the data are in favor of a null-hypothesis compared to the alternative hypothesis, we computed the null/alternative Bayes Factor (BF01). The Bayes Factor is a continuous likelihood-ratio statistical index, whose strength in supporting the null over the alternative hypothesis can be graded as follows: “Inconclusive” – BF01 = 0.33–3; “Substantial” – BF01 = 3–10; “Strong” – BF01 = 10–30; “Very strong” – BF01 = 30–100; and “Decisive” – BF01 >100 (Jarosz & Wiley, 2014; Morey & Rouder, 2018).

Furthermore, with very large sample sizes approaching the size of the population—in which theoretically any difference will be significant—statistical tests might have sufficient power to detect even tiny effects, often practically and theoretically irrelevant. In this connection, going further than the simple rejection of the null hypothesis of no effect, we tested for the presence of a meaningful effect, performing an equivalence analysis with the TOST procedure (Lakens, 2017; Lakens, 2018). A composite null hypothesis is formulated in this statistical approach, which states that a difference is at least as extreme as a threshold value expressed in standardized effect size. We used a small effect size (Cohen’s *d* ± 0.20) for the equivalence benchmarks in this study. The alternative hypothesis H1: (−0.20 < *d* < 0.20) of a true effect lying within the equivalence bounds, thus close enough to zero that the compared means can be considered practically similar, is set against the composite null hypothesis H0: (*d* ≤ −0.20 ∪ *d* ≥ 0.20) of a true effect large enough to be worth examining. It should be concluded that the observed values for the Home and Away teams are statistically equivalent if the composite null hypothesis can be rejected in two one-sided tests (TOST procedure).

As an additional set of analyses, to test for possible effects of Division and Country along with their interaction effect on Home advantage and Referee bias variables, we entered within-match differences as dependent variable into a 2 × 5 ANOVA model, with two between-matches factors: Division (two levels) and Country (five levels).

Statistical analyses of the present study were performed using R programming language (R Core Team, 2020).

## Results

**Figure 1:**
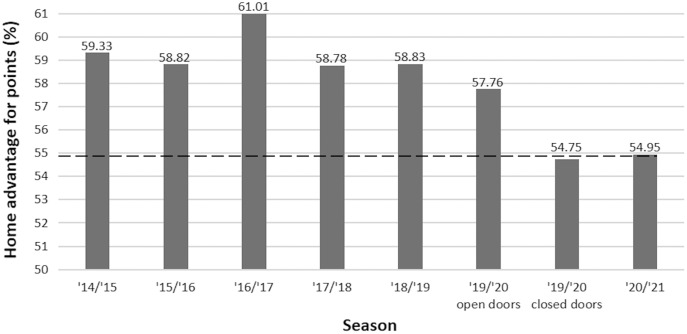
Home advantage for points season by season. The dashed line corresponds to the value observed in the 2020–21 season, to facilitate the comparison with the values of previous seasons. In the portion of the 2019–20 season behind closed doors, France is absent as both first and second divisions were not resumed after the lockdown.

The overall comparison for matches outcomes showed a statistically significant difference between the observed distribution in 2020–21 season and the expected frequencies calculated on the last five complete seasons without attendance restrictions (Chi-square = 37.42, df = 2, *p* < 0.001). Inspection of Pearson’s residuals within each cell showed that the observed frequency of home victories was significantly lower (*z* = −3.56, *p* < 0.001; 1/BF01 > 100, “Decisive” support to H1) and the observed frequency of away victories was significantly higher (*z* = 4.95, *p* < 0.001; 1/BF01 > 100, “Decisive” support to H1), compared to the expected distribution. The season by season analyses revealed a significant difference between the distributions of matches outcomes of the 2020–21 season and each season with spectators, but no difference with the portion of the 2019–20 season without spectators (see Table 2). The resulting values of the home advantage for points are 54.95% in the 2020–21 season, and 59.36% for the last five complete seasons without attendance restrictions; the home advantage values season by season are reported in Fig. 1.

**Table 2:** Comparison between expected and observed frequencies of matches’ outcomes.

	Home victories	Ties	Away victories	*N*
2020/21^a^	1,592	1,065	1,241	3,898
2019/20 (closed doors)	349	216	276	841
Standardized Pearson’s residuals (*z*)	0.351	−0.970	0.553	χ^2^_(2)_ = 0.97
2019/20 (open doors)	1,235	794	832	2,861
Standardized Pearson’s residuals (*z*)	**1.915**	0.392	**−2.428**	**χ^2^_(2)_ = 6.33**
2018/19	1,700	1,084	1,081	3,865
Standardized Pearson’s residuals (*z*)	**2.802**	0.714	**−3.721**	**χ^2^_(2)_ = 14.60**
2017/18	1,782	1,062	1,144	3,988
Standardized Pearson’s residuals (*z*)	**3.448**	−0.692	**−3.046**	**χ^2^_(2)_ = 13.62**
2016/17	1,863	1,062	1,063	3,988
Standardized Pearson’s residuals (*z*)	**5.256**	−0.692	**−5.059**	**χ^2^_(2)_ = 33.99**
2015/16	1,747	1,131	1,110	3,988
Standardized Pearson’s residuals (*z*)	**2.664**	1.029	**−3.886**	**χ^2^_(2)_ = 15.45**
2014/15	1,757	1,147	1,084	3,988
Standardized Pearson’s residuals (*z*)	**2.888**	1.423	**−4.533**	**χ^2^_(2)_ = 20.75**

**Notes:**

a The 2020/21 season acts as a reference outcome for all subsequent seasons’ frequencies. The standardized Pearson residuals for the 2020/21 season are not reported because they are simply the reciprocal of those calculated in the comparative season.

Values highlighted in bold are statistically significant (*p-values* < 0.05).

**Figure 2:**
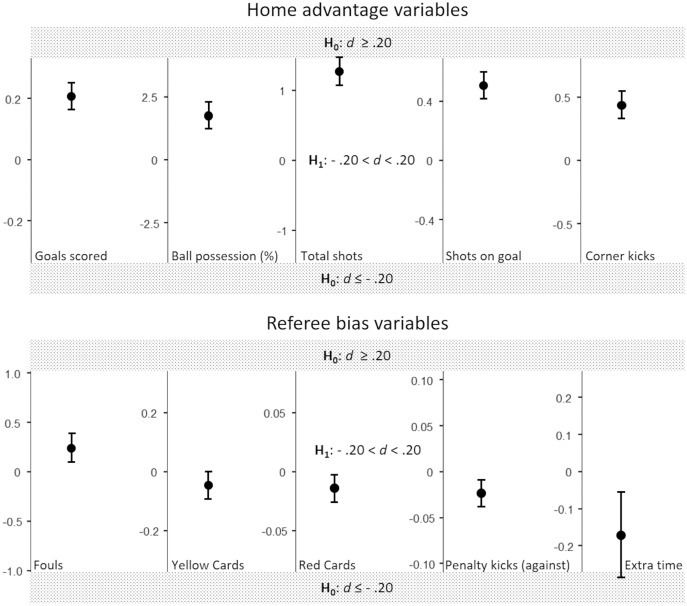
Illustration of the TOST test for home advantage and referee bias variables. The plot shows the observed mean differences, 95% confidence interval bars, and the equivalence bounds expressed in raw scores units. If the 95% confidence interval does not include the equivalence bounds, statistical equivalence is reached.

The results for the performance-related home advantage variables and the referee bias variables are reported in Table 3. As for the home advantage variables, a statistically significant overall advantage for the home team emerged. Nevertheless, the strength of the differences between home and away teams was generally small (0.09 < Cohen’s *d* < 0.17), and the corresponding means can be considered statistically equivalent for all variables but the total shots (see Table 4 and Fig. 2). This variable, in particular, produced nonsignificant results on the TOST procedure’s upper bound (set to Cohen’s *d* 0.20; see Fig. 2); thus, the equivalence test was nonsignificant, therefore it cannot be rejected the null hypothesis that the true effect size of the positive difference in total shots between home and away teams is small, at least.

**Table 3:** Statistical tests between home and away teams for home advantage and referee bias variables.

	Descriptive statistics				Paired t-tests					
	Home		Away							
	M	SD	M	SD	*t*	df	*p*	ES	Bayes Factor (H_0_/H_1_)	
Home advantage variables^a^										
Goals scored	1.39	1.22	1.18	1.11	7.63	3,897	***	0.12	0.00	–
Ball possession (%)	50.88	10.26	49.12	10.26	5.36	3,897	***	0.09	0.00	–
Total shots	12.20	4.84	10.93	4.58	10.62	3,897	***	0.17	0.00	–
Shots on goal	4.36	2.51	3.86	2.27	8.94	3,897	***	0.14	0.00	–
Corner kicks	4.90	2.69	4.46	2.57	6.61	3,897	***	0.11	0.00	–
Referee bias variables^b^										
Fouls	13.50	4.06	13.26	4.11	2.88	3,897	*n.s*.	0.05	401.36	Decisive
Yellow cards	1.95	1.37	1.99	1.38	−1.59	3,897	*n.s*.	−0.03	15.13	Strong
Red cards	0.09	0.30	0.11	0.33	−1.96	3,897	*n.s*.	−0.03	7.54	Substantial
Penalty kicks (against)	0.16	0.39	0.18	0.42	−2.52	3,897	*n.s*.	−0.04	2.11	Inconclusive
Extra time^c^	5.14	1.29	5.31	1.45	−2.44	1,484.1	*n.s*.	−0.12	0.81	Inconclusive

**Notes:**

a Calculated considering a one-sided null hypothesis. Expected positive Home-Away differences (Right tailed t-test).

b Calculated considering a one-sided null hypothesis. Expected negative Home-Away differences (Left tailed t-test).

c For this variable it was run an independent samples Welch’s *t*-test with adjusted degrees of freedom on the 1,567 matches whose result at 90’ had a difference of one goal (832 home team winning *vs*. 735 away team winning).

*** *p* < 0.001; *n.s*. = not significant.

Bayes Factor (H_0_/H_1_) = BF_01_, Null/Alternative hypothesis likelihood ratio; ES = Effect Size, Cohen’s *d*; M = Mean; SD = Standard Deviation.

**Table 4:** Two one-sided tests – TOST results for statistical equivalence.

	Lower bound H0: *d* ≤ −0.20 *vs*. H1: *d* > −0.20	Upper bound H0: *d* ≥ 0.20 *vs*. H1: *d* < 0.20	Statistical equivalence
Home advantage variables			
Goals scored	*t* = 20.12 (df = 3,897)***	*t* = −4.85 (df = 3,897)***	X
Ball possession (%)	*t* = 17.85 (df = 3,897)***	*t* = −7.12 (df = 3,897)***	X
Total shots	*t* = 23.10 (df = 3,897)***	*t* = −1.87 (df = 3,897) *n.s*.	
Shots on goal	*t* = 21.43 (df = 3,897)***	*t* = −3.55 (df = 3,897)**	X
Corner kicks	*t* = 19.10 (df = 3,897)***	*t* = −5.87(df = 3,897)***	X
Referee bias variables			
Fouls	*t* = 15.37 (df = 3,897)***	*t* = −9.60 (df = 3,897)***	X
Yellow cards	*t* = 10.90 (df = 3,897)***	*t* = −14.08 (df = 3,897)***	X
Red cards	*t* = 10.52 (df = 3,897)***	*t* = −14.45 (df = 3,897)***	X
Penalty kicks (against)	*t* = 9.96 (df = 3,897)***	*t* = −15.01 (df = 3,897)***	X
Extra time	*t* = 1.49 (df = 1,484.1) *n.s*.	*t* = −6.38 (df = 1,484.1)***	

**Notes:**

** *p* < 0.01.

*** *p* < 0.001; *n.s*. = not significant.

The statistical equivalence criterion is met when the composite null hypothesis H0: (*d* ≤ −0.20 U *d* ≥ 0.20) can be rejected in two one-sided tests, in which the null hypothesis is the *presence* of a true effect, defined by standardized differences (Cohen’s *d*) of *d* ≤ −0.20 (Lower Bound) or *d* ≥ 0.20 (Upper Bound), and the alternative hypothesis is the *absence* of an effect larger than these equivalence bounds.

As for the referee bias variables, there were no statistically significant differences between home and away teams for any of them, with substantial to decisive support to the null hypothesis for fouls, yellow cards, and red cards (see Table 3). For all variables, the means for home and away teams were statistically equivalent, with the only exception of extra time, where a negative small effect cannot be excluded (nonsignificant results on the lower bound set to Cohen’s *d* ≤ −0.20; see Table 4 and Fig. 2).

The results of the additional set of analyses for Division and Country showed no main effects of Division (all *p* values *≥* 0.069, and all BF01 ≥ 6.39) nor any interaction with Country (all *p* values *≥* 0.262, and all BF01 > 100) for all the Home advantage and Referee bias variables. As for the Country, statistically significant main effects emerged only for Corner kicks (*F*(4,3888) = 2.66, *p* = 0.031), Fouls (*F*(4,3888) = 3.75, *p* = 0.005), and Penalty kicks (*F*(4,3888) = 3.80, *p* = 0.004); however, although significant, the differences among countries were not supported by the Bayes factors (Corner kicks BF01 = 174.56, “Decisive” support to H0; Fouls BF01 = 13.38, “Strong” support to H0; Penalty kicks BF01 = 18.41, “Strong” support to H0).

## Discussion

In the present study, we examined the statistics of a complete football season with attendance restrictions due to the COVID-19 pandemic, in order to investigate how the absence of spectators influence the home advantage and the subconscious referee bias in the first and second divisions of the top five ranked European countries. Based on previous studies, we hypothesized a significant reduction of the home advantage with respect to previous seasons with spectators, a slight occurrence of the home advantage (due to the permanence of learning and travel factors), and no occurrence of the referee bias. The results support our hypotheses, providing more solid empirical evidence to the results initially observed in the 2019/20 season after the interruption because of the pandemic.

The majority of studies conducted on the 2019/20 season suggests a reduction – but not the elimination – of the home advantage (*e.g*., Bryson et al., 2021; Hill & Van Yperen, 2021; Jimenez Sanchez & Lavin, 2021; McCarrick et al., 2021; Scoppa, 2021; Sors et al., 2021), with some exceptions in which no reduction was found (*e.g*., Almeida & Werlayne, 2021), or—at the other extreme—no home advantage was observed at all (*e.g*., Tilp & Thaller, 2020). These apparently conflicting results can be attributed to the different leagues examined, to the unbalanced home/away schedule of the matches considered in the analyses (as only a portion of the season was played behind closed doors), and to the relatively limited number of matches. In the present study, we tried to overcome these issues, analyzing almost 4,000 matches from 10 complete leagues from five different countries. In accordance with the majority of previous studies, our results confirm a reduction of the home advantage. Similar to Sors et al. (2021), we found that the home-away difference of gained points decreases from around 20% points (roughly 60–40% with spectators) to around 10% points (roughly 55–45% with attendance restrictions). As for the performance-related home advantage variables, the analyses revealed significant differences in favor of the home teams also in absence of spectators, however these differences are mainly due to the large sample size. Indeed, the TOST analyses revealed meaningless effect sizes for four out of five variables, with the exception of total shots, for which a small effect size was observed and the statistical equivalence hypothesis was rejected. Overall, the results indicate that, in absence of spectators, the advantage for the home teams is reduced, however a certain degree of home advantage still persists.

Among the factors driving the home advantage, in normal conditions the referee bias plays an important role. However, the absence of spectators seems to eliminate this bias, with a substantial to decisive evidence in favor of null effects for fouls, yellow and red cards, and less clear results (but still non-significant home-away differences) for penalties and extra time. Similar to previous studies on matches behind closed doors (*e.g*., Bryson et al., 2021; Hill & Van Yperen, 2021; McCarrick et al., 2021; Scoppa, 2021; Sors et al., 2021), the absence of the social pressure normally exerted by spectators seems to influence referees in a positive way, making them fairer and less prone to make disparities between home and away teams. Consequently, the absence of referee bias is likely to contribute to the reduction of the home advantage observed when comparing matches with and without spectators, while the remaining quota of home advantage still observed in matches with attendance restrictions should be explained as the consequence of other factors.

The factors determining the home advantage have been summarized by Courneya & Carron (1992). According to their model, the game location factors (crowd, learning, travel, and rules) would affect the critical psychological states of competitors, coaches and officials, and consequently their behaviors and performances. Applying this model to our study, it is reasonable to hypothesize that the absence of the crowd—which would determine an absence of support for the home teams and a reduced pressure for the away teams—affected the psychological states of players and coaches (empirical support to this hypothesis was recently provided by Leitner & Richlan, 2021b). Similarly, the absence of pressure exerted by the crowd would influence the psychological states of the referees as well. Consequently, the critical behaviors of home and away teams’ members would be negatively *vs*. positively affected by crowd absence, respectively; in addition, the behavior (*i.e*., decisions) of the referees would be fairer (thus less advantageous for the home teams). Then, the combination of these effects would determine a reduction of the home advantage in absence of spectators, compared to regular matches with spectators. Notably, the remaining quota of home advantage in matches behind closed doors is not surprising and should be attributed to the other game location factors described in the model, in particular learning and travel. This is consistent with the observation that in national teams matches—in which learning and travel factors are comparable for home and away players—the home advantage disappears in absence of spectators (Sors et al., in press).

When investigating for potential effects of country and league level (first *vs*. second division), no meaningful differences emerged for any of the home advantage and referee bias variables included in the study. This indicates that the observed results are quite stable among the investigated countries, as well as between the first and second divisions within them. Although these results seem quite reliable, their validity cannot be generalized to all countries. Indeed, the present study focused on a limited number of countries, and future studies should therefore investigate the same phenomena also in different ones. In this regard, it would be of particular interest to examine whether similar effects occurred also in other continents, as well as in specific regions such as the Balkan area, where it is known that the home advantage is higher than the average (*e.g*., Pollard & Gomez, 2014).

A limitation of the present study is that a direct comparison with previous seasons was made exclusively for the distribution of matches outcomes, but not for the performance-related and referee bias variables; future studies should consider extending the comparison also to these variables, to gain further insights on the two phenomena. Another limitation of the study is that it consists in a “natural experiment”, which did not allow to control/manipulate the variables. Indeed, there was a small number of matches with a (very) limited attendance. We decided to include all matches in the analyses, as it was fundamental to have a fully balanced home/away schedule; we cannot exclude that the results may be even stronger excluding matches with a limited attendance. Another variable that was not possible to control is the video assistant referee (VAR). Indeed, during the period analyzed in the present study, the VAR was introduced in different seasons depending on the country/league (and French second division does not have it yet). Although we did not compare the referee bias in matches with and without attendance restrictions, we cannot exclude that, when comparing the home advantage in matches with and without such restrictions, the referee bias might have differently contributed to the home advantage depending on the absence/presence of the VAR. Finally, the expression “referee bias”, although normally used to describe the favoritism of referees towards the home teams, might also reflect the behaviors of home and away players in presence of spectators (*e.g*., more offensive attitude of home players, aggressive reaction of away players to booing). Thus, the observed absence of the referee bias in matches with attendance restrictions might be due not only to the absence of social pressure on the referees (Sors et al., 2019; Unkelbach & Memmert, 2010), but also to potential differences in players’ behaviors.

The results of the present study further highlights the relevant contribution of spectators to matches outcomes. From a practical perspective, football clubs should be aware of the significant impact of their fans, and should therefore adopt effective marketing strategies to maximize—obviously net of restrictions—the attendance at the home venue (*e.g*., discounts for bringing a friend/relative). Moreover, other strategical initiatives could be proposed to motivate fans to attend also away matches (*e.g*., free/discounted transportation), in order to at least partially contrast the influence of the opposite fans.

## Conclusions

In conclusion, the COVID-19 pandemic and its related restrictions gave us the chance to investigate how the absence of spectators modify the outcomes of football matches, as well as performance parameters and referees’ behaviors. In normal conditions, the home teams have a clear advantage due to several factors, including the support of their fans and the referee bias in their favor. Our findings demonstrate that the absence of spectators significantly reduces the home advantage and eliminates the referee bias, providing more solid evidence compared to previous literature. The persistence of the home advantage—although significantly reduced—indicates that other factors such as the familiarity with local conditions and the travel fatigue play an important role and somehow influence the players’ behavior and the subsequent outcomes. Conversely, the absence of referee bias suggests that it mainly derives from the pressure normally exerted by spectators.

## Supplemental Information

10.7717/peerj.13681/supp-1 Supplemental Information 1 Raw data.

## Additional Information and Declarations

### Data Availability

The following information was supplied regarding data availability: The raw data is available in the Supplemental File.
